# A Novel Mobile Health App to Educate and Empower Young Adults With Type 1 Diabetes to Exercise Safely: Prospective Single-Arm Pre-Post Noninferiority Clinical Trial

**DOI:** 10.2196/68694

**Published:** 2025-08-22

**Authors:** Vinutha Beliyurguthu Shetty, Rachel Lim, Shaun Teo, Wayne H K Soon, Heather C Roby, Alison G Roberts, Grant J Smith, Paul A Fournier, Timothy W Jones, Elizabeth A Davis

**Affiliations:** 1 Department of Endocrinology, Perth Childrens Hospital, 15 Hospital Avenue, Nedlands, WA, Perth City, 6009, Australia, 61 864565020 ext 65020; 2 Division of Paediatrics within the Medical School, The University of Western Australia, Perth City, Australia; 3 Children's Diabetes Centre, The Kids Research Institute Australia, West Perth, Australia; 4 School of Allied Health (Exercise Science), Murdoch University, Perth City, Australia; 5 Department of Exercise Science and Health, School of Human Sciences, The University of Western Australia, Perth City, Australia

**Keywords:** mobile health app, exercise, acT1ve, type 1 diabetes, young people, blood glucose level

## Abstract

**Background:**

A novel mobile health (mHealth) app “acT1ve,” developed using a co-design model, provides real-time support during exercise for young people with type 1 diabetes (T1D).

**Objective:**

This study aimed to demonstrate the noninferiority of acT1ve compared with “treatment as usual” with regard to hypoglycemic events.

**Methods:**

Thirty-nine participants living with T1D (age: 17.2, SD 3.3 years; HbA_1c_: 64, SD 6.0 mmol/mol) completed a 12-week single-arm, pre-post noninferiority study with a follow-up qualitative component. During the intervention, continuous glucose monitoring (CGM) and physical activity were monitored while participants used acT1ve to manage exercise. CGM data were used to assess the number of hypoglycemic events (<3.9 mmol/L for ≥15 minutes) in each phase. Using a mixed effects negative binomial regression, the difference in the rates of hypoglycemia between the preapp and app-use phases was analyzed. Participants completed both a semistructured interview and the user Mobile Application Rating Scale (uMARS) questionnaire postintervention. All interviews were audio-recorded for transcription, and a deductive content analysis approach was used to analyze the participant interviews. The uMARS Likert scores for each subscale (engagement, functionality, esthetics, and information) were calculated and reported as medians with IQRs.

**Results:**

The rates of hypoglycemia were similar for both the preapp and app-use phases (0.79 and 0.83 hypoglycemia events per day, respectively). The upper bound of the CI of the hypoglycemia rate ratio met the prespecified criteria for noninferiority (rate ratio=1.06; 95% CI 0.91-1.22). The uMARS analysis showed a high rating (≥4 out of 5) of acT1ve by 80% of participants for both functionality and information, 72% for esthetics, and 63% for overall uMARS rating. Content analysis of the interview transcripts identified 3 main themes: “Provision of information,” “Exercising with the App,” and “Targeted Population.”

**Conclusions:**

The mHealth app “acT1ve,” which was developed in collaboration with young people with T1D, is functional, acceptable, and safe for diabetes management around exercise. The study supports the noninferiority of acT1ve compared with “treatment as usual” with regards to hypoglycemic events.

## Introduction

Children with type 1 diabetes (T1D) diagnosed before the age of 10 years have a 30-fold higher risk of coronary heart disease in early adulthood [1], and despite advances in care, life expectancy is reduced by 12 to 16 years [1]. Cardiovascular disease is the most common cause of shortened life expectancy in T1D and is clearly linked to key modifiable factors, including exercise [2-6]. In this respect, there is evidence that regular exercise has the potential to improve clinical outcomes and reduce cardiovascular morbidity and mortality in people with T1D [2]. Indeed, the amount of exercise an adult with T1D undertakes is inversely related to glycated hemoglobin A_1c_ (HbA_1c_) levels, BMI, the prevalence of diabetic ketoacidosis, retinopathy, microalbuminuria, hypertension, and dyslipidemia [3]. Furthermore, physically active children and adolescents with T1D display better glycemic levels, endothelial function, body composition, neurocognitive, and psycho-behavioral function [4-6].

Despite the many benefits of regular exercise, many people with T1D do not meet the current physical activity recommendations [7], especially adolescents with T1D who are less active than their peers without T1D [8]. Apart from the risk of exercise-mediated hypoglycemia, inadequate patient and health care provider knowledge about exercise management are barriers to an active lifestyle in young people living with T1D [9,10], and programs designed to increase physical activity have so far been ineffective [11].

Although detailed exercise recommendations have been provided by key professional societies and organizations for the prevention of exercise-mediated hypoglycemia [12-14], these recommendations can be challenging to follow and are often found in medical journals that are not readily accessible to the general T1D community and clinicians alike. A recent survey conducted by our team led us to propose that providing exercise guidelines in a mobile health (mHealth) app would be useful as a decision-support aid around exercise management for adolescents and young adults with T1D [10]. Indeed, mHealth apps that track diabetes-related health information, provide education, and connect patients to support systems could potentially facilitate patients’ self-management and improve diabetes-related outcomes. Increasingly, patients with diabetes have thus been using mHealth apps to assist with their diabetes self-management [15-19]. Currently, there are no commercially available apps that specifically support diabetes self-management and provide individualized information around exercise in young people living with T1D. The Diactive-1 app has been recently developed by a team of researchers from the University of Turin, Italy. This app is being tested to explore the potential benefits for various aspects of T1D management, including personalized physical exercise [20].

We recently developed in collaboration with young adults with T1D and the digital health company Curve Tomorrow, the novel mHealth app, “acT1ve” [21]. The app was based on recent exercise guidelines consensus [12-14] and developed following a user-centered design process that engaged end-users to ensure app effectiveness [22]. In a recent pilot trial, acT1ve was found to be informative, functional, and acceptable with high user satisfaction, making it a promising intervention for exercise management [23]. Thereafter, improvements to the app were made based on the feedback gathered from the pilot trial. However, a component that has yet to be investigated relates to the safety surrounding app usage. This is an important element to be addressed, given that the process of gaining Australian regulatory body approvals to allow for the app to reach the market requires the app to comply with the essential principles relating to safety. For this reason, the primary objective of the current study was to conduct a clinical trial to test the safety of acT1ve at providing real-time support for young people with T1D during exercise in a free-living setting by showing the noninferiority of acT1ve compared with “treatment as usual” with regards to hypoglycemic events. The secondary objectives of the study were to explore the overall usability, acceptability, and experience of acT1ve over a 4-week period, and to gather qualitative feedback on the user experience of acT1ve and changes in exercise behavior and trends.

## Methods

### Design

The project adopted a single-arm pre-post noninferiority study design and was performed under free-living conditions in adolescents and young adults with T1D from September 2020 to December 2021. The CONSORT (Consolidated Standards of Reporting Trials) checklist is provided in Checklist 1.

### Study Participants

Forty-two individuals (males and females) were recruited to participate in the study (Table 1). Inclusion criteria included age (12‐25 years), T1D diagnosis (>6 months), insulin therapy (multiple dose insulin [MDI] regimen or continuous subcutaneous insulin infusion [CSII]), being able or willing to perform regular exercise (≥2 sessions per week), smartphone ownership either Android or iPhone, and English competency. Exclusion criteria were reduced cognitive capacity that impaired the ability to consent/assent and non-English speaking individuals. Participant recruitment was performed through the Western Australian Children’s Diabetes Database and approached via email or phone, or by a researcher face-to-face when they attended Perth Children’s Hospital diabetes clinics. Flyers were provided to the PCH Diabetes Clinical Service, and the study was advertised on Diabetes community organizations’ websites, social media, and their newsletters.

**Table 1.** Participant demographics.

Demographics	Participants (n=39)
Sex, n (%)	
Male	19 (49)
Female	20 (51)
Age (years), mean (SD)	17.2 (3.3)
HbA_1c_^a^ (%), mean (SD)	7.9 (1.5)
HbA_1c_ (mmol/mol), mean (SD)	64.0 (6.0)
Insulin regime, n (%)	
CSII^b^	21 (54)
MDI^c^	18 (46)
T1D duration (years), mean (SD)	6.9 (1.2)
BMI (kg/m2), mean (SD)	22.6 (3.9)
Physical activity levels, median (IQR)	
Exercise intensity (METs)^d^	5.4 (4.8, 6.4)
Weekly exercise duration (min)	80.9 (35.4, 163.9)
History of mobile app use, n (%)	
General exercise-based	8 (21)
Diabetes-specific exercise-based	0 (0)
Type of mobile device used for acT1ve app, n (%)	
Apple iOS	31 (79)
Android	8 (21)

a HbA_1c_: glycated hemoglobin A1c.

b CSII: continuous subcutaneous insulin infusion.

c MDI: multiple daily injections.

d MET: metabolic equivalent of task.

Previously analyzed data collected as part of a longitudinal randomized controlled trial [24], in a sample of 12 to 25-year-old participants, along with data collected in a pilot study [23], were used to inform parameters of the sample size power calculation. Given the (1) estimated rate of hypoglycemic events<3.9 mmol/L was approximately 0.3 events per 24 hours (2) a dispersion parameter (theta) ranging between 2 to 2.5 and (3) correlation between rates of hypoglycemic events measured longitudinally ranging between 0.5‐0.7, a noninferiority limit was identified based on discussions with clinicians and was set at a 50% increase in event rate. Based on an intervention duration of 4 weeks, with expected equivalent rates, and specifying the more conservative of the above parameters (theta=2 and correlation=0.5), 1000 simulations were conducted, indicating that a sample of 40 participants would provide over 85% power for the upper boundary of a 95% CI to fall below the noninferiority limit.

### Study Flow

#### Overview

**Figure 1.**
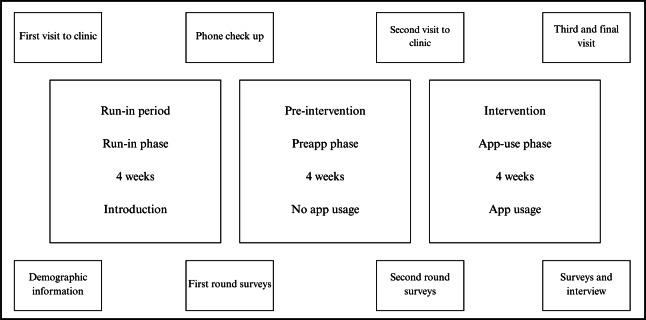
Schematic representation of the study design.

All participants were required to complete the study intervention over a 12-week period during school terms to ensure minimal variation for both children and adolescent participants. Each 12-week period was split into 3 phases, with each phase lasting 4 weeks (Figure 1). The participants attended our research facility on 3 occasions throughout the study: at the start of the run-in phase (Week 1), at the start of the preapp phase (Week 5), and at the end of the app-use phase (Week 12). Participants’ glucose levels during the study period were monitored using a Dexcom G6 continuous glucose monitoring system (CGM), and physical activity events were recorded with a Garmin Forerunner Activity-monitoring watch or, if the activity watch was not worn during exercise, a self-reporting logbook. Participants were also required to complete a series of questionnaires in Weeks 5, 8, and 12. During each phase, participants wore the CGM throughout the study period and activity monitoring watch, only while exercising.

#### Run-in Phase

The 4-week run-in phase was used to familiarize the participants with the CGM and activity watch. During the first visit, demographic and descriptive characteristics of participants were collected, which included: age, sex, duration of diabetes, HbA_1c_ level, insulin therapy, and exercise patterns. Thereafter, participants were sent home and instructed to familiarize themselves with the CGM and activity watch. Physical activity events and data from each participant’s watch were linked and tracked through their individual Garmin Connect online account. The CGM data were monitored through the Dexcom Clarity (Dexcom, Inc) online software. The participants were also reminded to record their activity in their paper logbook if it was not recorded on the activity watch and to log the reasons for any gaps in physical activity participation. This routine was set up to ensure smoother data collection during the subsequent preapp phase. Activity watch and CGM were monitored weekly to ensure that these pieces of equipment were functional and to ensure the consistent collection and storage of the data through the software used.

#### Preapp Use Phase

During the preapp phase, participants continued to wear the CGM and activity watch as per the run-in phase and were encouraged to exercise and follow their usual blood glucose management routine. A requirement for the study was that participants were currently or willing to exercise a minimum of 2 times a week. For already active participants, they were asked to go about their usual exercise routines. Participants who were not currently exercising 2 times a week were encouraged to do so as part of the study. We defined exercise duration of 5 minutes or more as a session of exercise. This phase was primarily aimed at collecting the data to be compared with that of the intervention phase (app-use phase). Participants were contacted in the event that gaps in the data collection were identified. Participants were also asked to complete their first set of questionnaires online in week 5, with the links to the questionnaires being sent to their respective emails.

#### App-Use Intervention Phase

At the commencement of the app-use phase, participants returned to the research facility to have the acT1ve app downloaded on their smartphones and to complete their end of preapp series of questionnaires. Then, the participants’ profiles were set up within the app, and they were instructed to follow the in-built onboarding process, which guided them through the different sections and functions of acT1ve app before leaving the research facility. Participants were instructed to use the app for 4 weeks to assist them with their exercise-related diabetes management. Participants continued to use the CGM and the activity watch as per the preapp phase. Throughout the App-use phase, participant data were monitored on a regular basis, and participants were contacted should there be any missing data. Data regarding participants’ use of the app was recorded on REDCap (Research Electronic Data Capture) [25]. During their final visit, at the end of the app-use phase, participants were assisted in deleting the acT1ve app from their device before completing their final set of questionnaires. In addition, participants completed the user Mobile Application Rating Scale (uMARS) questionnaire [26] and participated in a semi-structured face-to-face interview to provide feedback on their experiences of using the app.

### acT1ve Mobile App

The acT1ve app uses an exercise advisor algorithm developed in-house that is based on recently published evidence-based guidelines [23] and provides 240 possible pathways, which are dependent on user inputs [23]. Participants are prompted to answer questions about the type, intensity, and duration of the physical activity they are about to complete, time elapsed since the last insulin bolus, and their current blood glucose levels. This information is then used to provide personalized insulin dosing and carbohydrate advice for exercise lasting up to 60 minutes. In addition, acT1ve provides more information on hypoglycemia treatment, pre- and postexercise insulin and carbohydrate advice, and an educational food guide that highlights the importance of low and high glycemic index (GI) foods in the context of exercise management.

### Continuous Glucose Monitoring (CGM)

Continuous glucose monitoring data were collected using the Dexcom G6 sensor. Each participant had their own personal Dexcom Clarity account where their data were collected and stored in 5-minute intervals. At the end of the participant’s study period, a member of the research team downloaded the CGM data in a CSV Excel (Microsoft) file, and all data were deidentified for analysis.

### Monitoring of Physical Activity

Participants were assigned their individual Garmin account, with instructions provided on how to pair their Garmin Forerunner 735XT activity monitoring watch to the Garmin Express mobile app. Physical activity events were monitored using the Garmin Connect desktop application. In the event where an activity could not be registered on the activity watch, participants were instructed to log such an activity in a paper diary. Physical activity events recorded on the activity watch were downloaded at the end of each study phase. To determine if any exercise bout is a ‘true’ event, the duration of the exercise bout recorded by the activity watch had to be ≥5 minutes in duration. All physical activity events that were <5 minutes were considered accidental/error and were excluded from the final analysis. Metabolic equivalent of tasks (METs) was used as a measure of exercise intensity. One MET is defined as the amount of oxygen consumed while sitting at rest and equates to an oxygen consumption rate of 3.5 ml/kg/min [27]. Classification of exercise intensity was based on the following MET levels as recommended by the American College of Sports Medicine guidelines: [28] light intensity activity (1.1‐2.9 METs); moderate intensity activity (3.0‐5.9 METs); and vigorous intensity activity (≥6.0 METs). The calculation of energy expenditure based on MET data depended on the method used to assess physical activity (activity watch or paper diary entries). When the energy expended during exercise was available from the activity watch, the following formula was used to calculate energy expenditure and thus exercise intensity (Energy expenditure calories = (MET level of activity x 3.5x Weight (kg) x minutes of activity)/200) [29]. In the absence of MET data, exercise intensity was calculated based on a compendium of predicted MET values for specific activities as outlined in the 2011 Compendium of Physical Activities [29].

### uMARS Questionnaire

The uMARS questionnaire [26] was used to evaluate acT1ve app. This tool is used to assess the overall quality of mHealth apps and provides a 20-item measure that includes four objective quality subscales, namely engagement, functionality, esthetics, and information quality, and 1 subjective quality subscale. A total quality score is obtained from the weighted average of the 44 subscales. Another subscale, consisting of 6 items, is added to measure users’ perceived impact of the evaluated app [26], where the details of the subscales have been described previously [23]. At the end of the app-use phase, the uMARS questionnaire was administered to the participants. Scores for the four objective subscales were determined by the mean score of each of its individual questions. The perceived impact and subjective quality of acT1ve for each participant were calculated by averaging the scores of their related questions but were not considered in the total quality score.

### Participant Interview

At the participants’ final study visit, they were asked to participate in a semistructured interview. The interview questions (Multimedia Appendix 1) were designed to gain an understanding of the participants’ experiences of using the app for exercise, their usability and acceptability of the app, overall experience, and any recommendations. The interviews were conducted by 3 researchers trained in qualitative interviewing techniques. Two of the interviewers were unknown to the participants. The third interviewer was a member of the project team known to the participants but was not in a senior position or involved with the participant in an ongoing capacity, either in research or their clinical care. After each interviewer conducted their first interview, the other interviewers listened to the recording to review interviewing methods for consistency. All interviews were audio-recorded for transcription and analysis.

### Outcomes

The primary outcome of the study was the rate of level 1 hypoglycemia (<3.9 mmol/L for ≥15 min) as collected by the CGM device during each study phase. The secondary outcomes were: incidence of level 2 hypoglycemia (<3.0 mmol/L for ≥15 min), time spent with sensor glucose levels between 3.9 and 10 mmol/L, time spent above target glucose range (>10 mmol/L), incidence of level 1 and 2 hypoglycemia or treated hypoglycemia during the subsequent 24 hours after exercise [1], overall perceived quality of acT1ve as measured by the uMARS questionnaire [26], changes in exercise patterns (ie, exercise frequency, duration, and intensity), and qualitative feedback relating to user experience of acT1ve.

### Statistical Analyses

#### Quantitative Analysis

To evaluate the noninferiority of acT1ve use over a 4-week period, we tested the null hypothesis that acT1ve treatment was not associated with a higher rate of level 1 hypoglycemic events, as defined above, than “treatment as usual” (preintervention phase). To accept the null hypothesis and conclude noninferiority of the intervention, the upper bound of the 95% CI of the ratio of the rate of hypoglycemic events in the intervention phase to the preintervention phase had to fall below the noninferiority limit of 1.5. Our primary analysis thus assessed the difference in the rate of level 1 hypoglycemia between the preapp and app-use phases. This was analyzed using a mixed effects negative binomial regression including a random effect for participant and a fixed effect for study phase (preapp and app-use phases). The dispersion parameter was estimated using maximum likelihood estimation approximating the integrals over the random effects with an adaptive Gaussian quadrature rule. The incidence rate ratio, along with its 95% CI, was calculated.

Percent time in ranges and continuous secondary outcomes were analyzed using a linear mixed model including a random effect for individual and a fixed effect for study phase (preapp and app-use phases). Percentages and medians IQRs (IQR: 25%-75%) were calculated for the monitoring of “true” and acT1ve app physical activity events recorded during both phases, each uMARS subscale, and total score. Means (pooled SDs) for exercise frequency, intensity, and duration across the respective study phases were analyzed, and the magnitude of change between phases for each of the exercise components was reported using Hedges *g* and interpreted as small (g=0.2), moderate (g=0.5), or large (g=0.8) [30]. Statistical significance for all quantitative analyses was set at *P*<.05.

#### Qualitative Analyses

A deductive content analysis approach [31] was used to analyze the participant interviews, as some questions were based on previously identified concepts from the pilot app trial study. Three researchers (ST, RL, and AR) worked independently to read and reread all transcripts to develop categories from the data. Researchers met to discuss categories and determine themes that encompassed the participant experience in relation to the research question.

### Ethical Considerations

The study was approved by the Child and Adolescent Health Human Research Ethics Committee (RGS0000003886), and all participants provided consent in accordance with the Child and Adolescent Health Human Research Ethics Committee, registered with the National Health and Medical Research Council’s Australian Health Ethics Committee. In addition, parental consent was also obtained for participants under the age of 18 years. All study data were deidentified. Participants were provided US $65 in cash at the end of their participation. The project is registered with the Australian New Zealand Clinical Trials Registry (ACTRN12620001066976).

## Results

### Demographics

**Figure 2.**
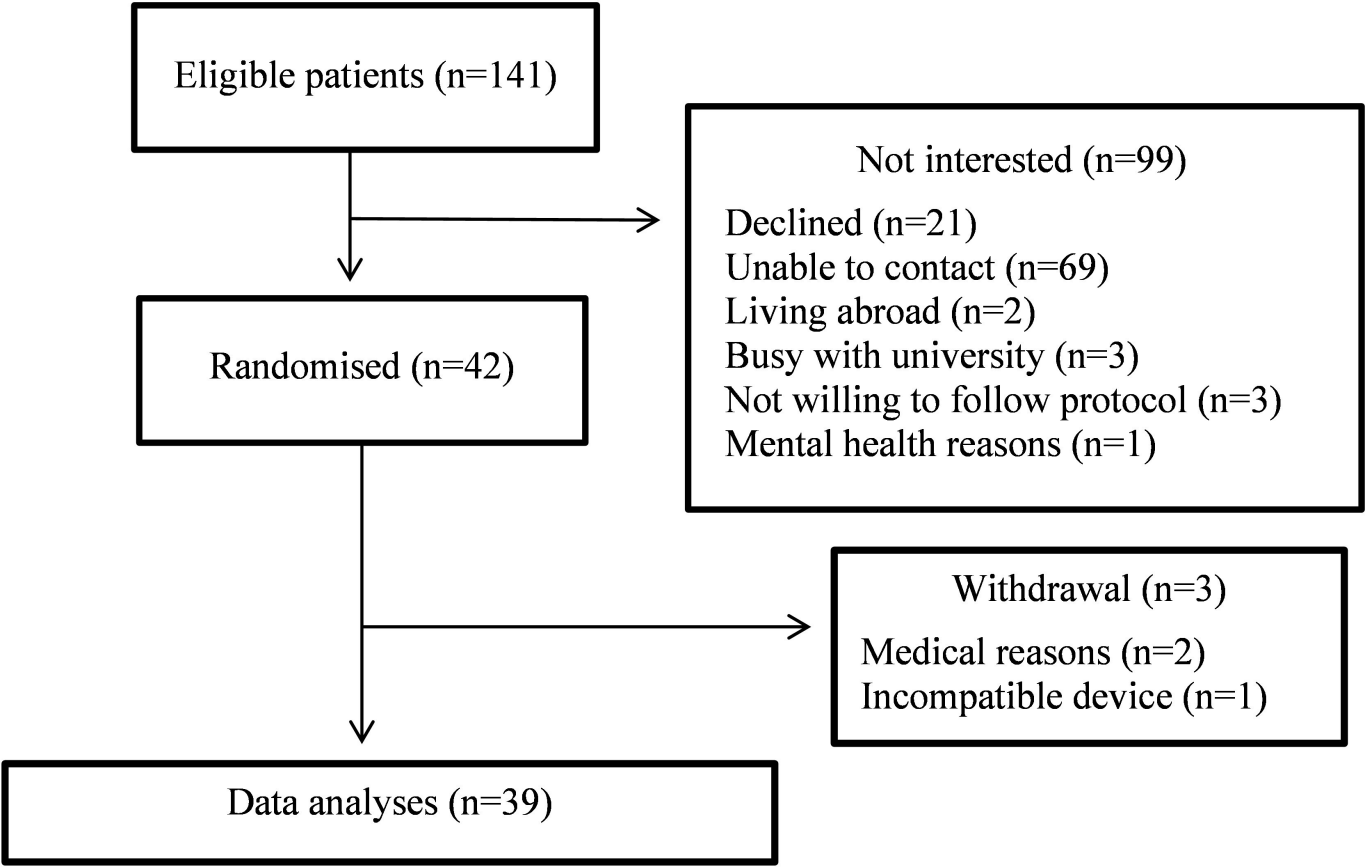
The CONSORT (Consolidated Standards of Reporting Trials) flow chart for participants in the trial.

As illustrated in Figure 2, 42 individuals (20 males and 22 females) consented and were enrolled in this study, with 39 included in the final analysis (demographics presented in Table 1). Three participants withdrew in the run-in period. Two participants were excluded because of acute health issues (1 had a viral infection followed by chronic fatigue, and the other had issues relating to mental health), and the data from 1 participant was excluded due to incompatibility between the mobile device and the Dexcom CGM app.

### Quantitative Results

#### Glycemic Outcomes

**Figure 3.**
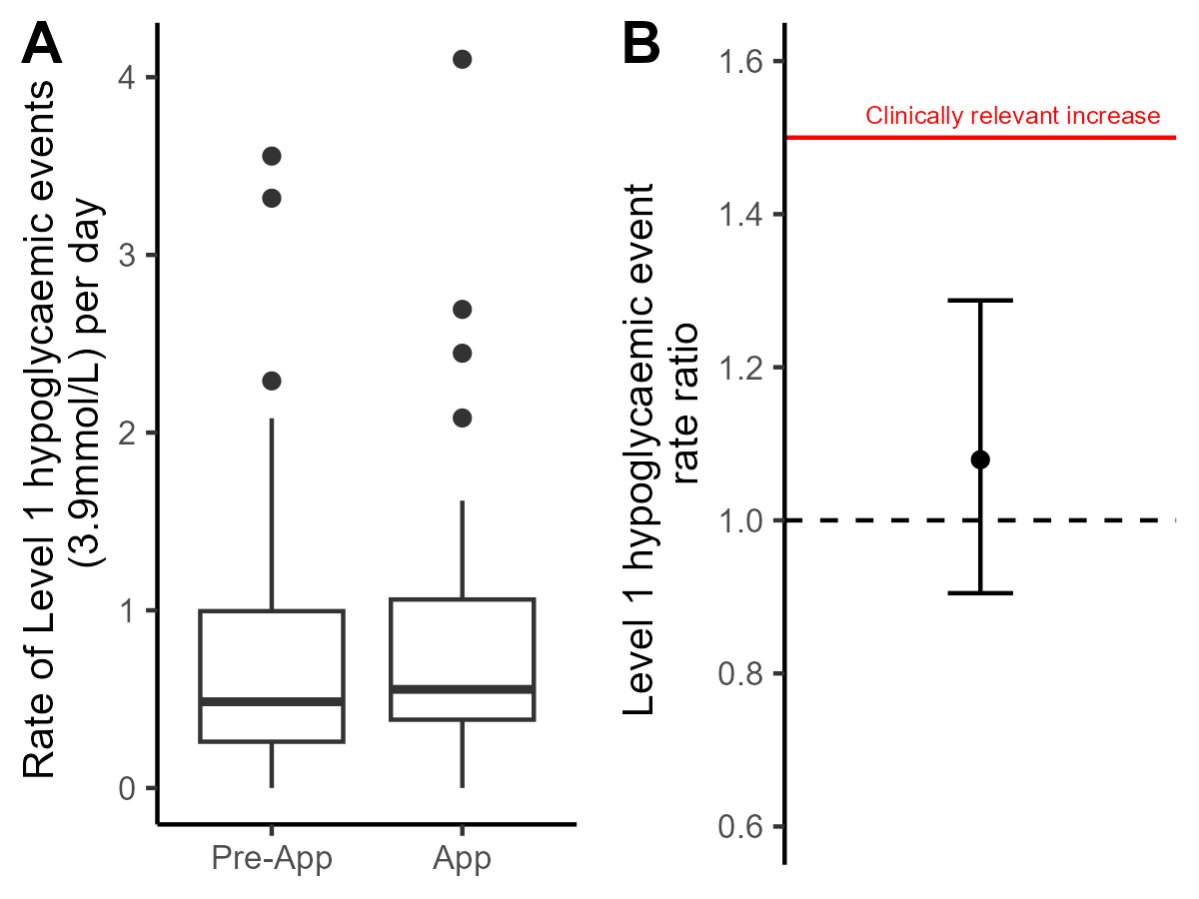
Change in hypoglycemia rate from preapp to app-use phase. (A) Box and whisker plot of Level 1 hypoglycemic events in preapp and app-use phases. (B) Change in rate from preapp to app-use phase presented as a rate ratio with 95% CIs. The red line represents the clinically meaningful increase in level 1 hypoglycemia events.

Similar rates of level 1 (0.79, SD 0.82, and 0.83, SD 0.84 events per day, respectively) and level 2 hypoglycemia (0.25. SD 0.48 and 0.24, SD 0.50 events per day, respectively) were observed for the preapp and app-use phases during the subsequent 24 hours after exercise. The upper bound of the confidence interval of the level 1 hypoglycemia rate ratio met the prespecified criteria for noninferiority (rate ratio=1.06, 95% CI 0.91-1.22; Figure 3A-B). The percentage of time spent within the target glucose range and the time spent above target glucose range during the subsequent 24 hours after exercise did not differ between preapp and app-use phases (Table 2).

Similar rates of level 1 and level 2 hypoglycemia, the percentage of time spent within the target glucose range, and the time spent above the target glucose range were observed during and 1 hour after exercise for the preapp and app-use phases (Table 3).

**Table 2.** Key glycemic metrics during 24 hours after exercise.

CGM^a^ metric	Preapp phase, mean (SD)	App-use phase, mean (SD)	*P* value
Time in range (%)	53.3 (17.4)	54.5 (16.7)	.27
Time high (%)	43.3 (18.9)	42.3 (18.1)	.35
Mean IGL^b^	10.1 (2.2)	10.0 (2.1)	.44
SD IGL^c^	3.78 (0.95)	3.80 (0.85)	.49

a CGM: continuous glucose monitor.

b IGL: interstitial glucose levels.

c SD IGL is the mean of all the SDs of all participants and reflects glycemic variability.

**Table 3.** Key glycemic metrics during/immediately after exercise.

CGM^a^ metric	Preapp phase, median (IQR)	App-use phase, median (IQR)	*P* value
Time spent <3.0 mmol/L (%)^b^	0 (0,0)	0 (0,1.67)	.12
Time spent <3.9 mmol/L (%)^b^	0.71 (0, 3.77)	1.65 (0, 8.35)	.25
Time spent 3.9‐10.0 mmol/L (%)^c^	53.7 (25.6, 69.3)	50.5 (32.6, 70.8)	.96
Time spent >10.0 mmol/L (%)^c^	45.0 (27.4, 72.8)	42.2 (22.9, 67.4)	.77
Mean SGL^d^ (mmol/L)^c^	10.3 (8.2, 11.9)	9.7 (7.9, 12.2)	.52
SD SGL^e^ (mmol/L)^b^	3.1 (2.6, 4.0)	3.0 (2.7, 3.9)	.91

a CGM: Continuous glucose monitor.

b *P* value calculated using the Wilcoxon signed rank.

c *P* value calculated using paired *t* test.

d SGL: sensor glucose levels.

e SD SGL is the mean of all the SDs of all participants and reflects glycemic variability.

#### Types of Exercise Events Recorded

For both the preapp and app-use phases, approximately 42% of the exercise activities participants engaged in were aerobic-based activities (ie, walking, jogging, running, and cycling). The other activities that participants engaged in were: (1) sport-specific (eg, team-based sports and archery); (2) water-based (eg, swimming, surfing, and rafting); (3) strength-based (eg, gym, weightlifting, and Pilates) and (4) nonstructured exercise (eg, gardening, housework, and school-based activities). These made up approximately 24%, 14%, 14%, and 7% of the activities participants engaged in across both the preapp and app-use phases, respectively.

#### Monitoring of Physical Activity Events and acT1ve App Use

The median frequency of “true” exercise events per week recorded via both the activity watch and paper diary was similar between Preapp (1.75; IQR 1.00‐3.00 events) and App-use phases (1.75; IQR: 1.25‐3.00 events). In the app-use phase, the median frequency of events per week was 1.5 (IQR: 0.75‐2), with no significant difference observed between ‘true’ exercise events and app-recorded events (*P*>.05).

#### Changes in Frequency, Intensity, and Duration of Exercise

There were no significant differences in total monthly exercise frequencies (10.74, SD 9.35 vs 10.33, SD 9.57; *g*=0.09), average exercise intensity (5.4, SD 2.4 vs 5.4, SD 2.6 METs; *g*=0.02), average exercise duration (46.6±31.3 vs 48.9±33.5 min; *g*=0.07) and total monthly exercise workload (3403.0, SD 4768.3 vs 2697.8, SD 2770 MET-min; *g*=0.18; all *P*≥.43) between preapp and app-use phases.

#### uMARS

**Figure 4.**
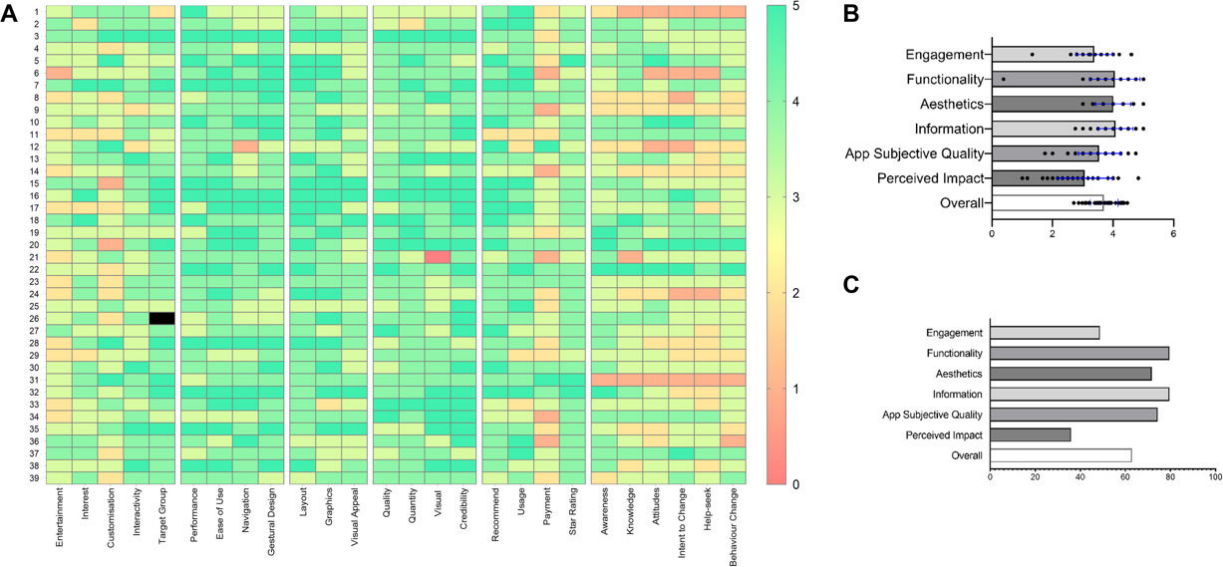
User Mobile Application Rating Scale (uMARS) questionnaire scores. 5A: Individual participant questionnaire response scores; 5B: Average quality scores for each uMARS category; and 5C: Percentage of participants rating a score of ≥4 (“good” or “excellent”) for each category.

The use of acT1ve app was associated with a uMARS total quality median score of 4 (IQR 3.1‐4.3; Figure 4), which corresponds to a “good” score for overall acceptability. The uMARS objective quality subscale scores (Figure 4) for engagement, functionality, esthetics, information, application quality, and perceived impact were 3.4 (IQR 3.0‐3.8), 4.1 (IQR 3.8‐4.7), 4.0 (IQR 3.7‐4.3), 4.0 (IQR 3.8‐4.5), 3.6 (IQR 3.3‐4.0), and 3.2 (2.5‐3.8), respectively.

### Qualitative Results

**Figure 5.**
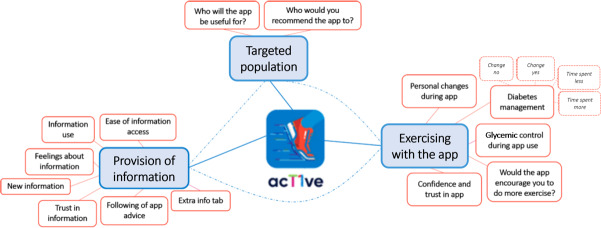
Identification of themes and subthemes from thematic interview analysis.

The interview analysis identified 3 main themes: “Provision of information”; “Exercising with the App”; and “Targeted Population” (Figure 5).

#### Provision of Information

The “more info” tab in the app contained information about hypo treatment, food guide, and pre- and postexercise advice. This information reflected the international guidelines and served as an educational toolbox for participants. The information tab was viewed by all participants, with comments including that the information was extensive and interesting, with some participants recommending that it needed important aspects to be highlighted by color or bold font. Many participants commented that once they had read the information, they felt that they did not need to refer back to the information on subsequent occasions. Some participants who had longstanding diabetes found the information interesting but not new, while others liked that it acted as a good refresher for information that they had forgotten.

*Most of the time there wasn’t quite so much planning involved because I didn’t have to like figure things out as much, I could just put in like my sugar levels, insulin levels as they were, and then get the information and just go*.

[Participant #23]

Participants felt they could trust the information as it had been provided by a reliable source and were happy that they could access it readily if needed.

*Yeah, but like it helped a lot. I have more confidence. It’s like actual information that has been put across that you can trust and use*.

[Participant #17]

#### Exercising With the App

Participants found the app easy to navigate, straightforward, and user-friendly, and liked the simplicity of the interface and how easy it is to navigate the app. One participant noted that,

*It was easy, like it’s just... the way they made it is like so simple, it like reminds you of like apps that have nothing to do with diabetes at all...*

[Participant #40]

All participants accessed the app for exercise at least once; however, more than half of the cohort did not use the app for their regular exercise. A plausible explanation for this may be that most participants who were recruited for the present study were already very active and had already determined a management plan for their routine exercise regimen that seemed to work. Some participants who followed the recommended advice from the app reported that the app suggested consuming more carbohydrates or reducing their insulin dose more than what they normally would. Despite this, many participants felt reassured by the information, as it indicated that they were performing the desired diabetes management strategies required during exercise performance.

Participants who used the app for new or spontaneous exercise felt comfortable following the recommendations and reported that following the recommendations resulted in reduced postexercise hypoglycemia and/or improved glycemia throughout the exercise or activity period.

*Yes, it was definitely easier and definitely gives me more self-confidence to know that what I’m doing, ... I’m like, ... more likely to be safer when riding and having, ...not having the risk of going low and having hypo...*

[Participant #19]

The use of the app did not result in an overall increase in exercise or activity; however, 5 participants commented that using the app may encourage them to do a new exercise.

#### Targeted Population

The app was not used to its full potential by most study participants, as those who exercise frequently were already confident in their management and either forgot to use it or felt it was an extra step to their preparation. Many participants used the app out of curiosity and because they had agreed to the study. All participants commented that this app would be better suited to those individuals with T1D, who were new to exercise, newly diagnosed, or who had fears around engaging in regular exercise [2].

*I think it would be good for people who have just gotten diagnosed because they wouldn’t really know, and they won’t have that much information, but I think the app would help them a lot and how to get them started with their sugar levels with activities*

[Participant #1]

Participants mentioned this app would also be beneficial for parents to increase their confidence when encouraging their child to become more independent with their diabetes management, or when their child was away from them.

## Discussion

### Principal Findings

Given that it is a requirement by several regulatory bodies to subject any new therapeutic tool to noninferiority testing to indicate that the tool in question is not worse when compared to “treatment as usual,” the aim of the current study was to test the safety of the novel mHealth app “acT1ve.” The study supports the noninferiority of acT1ve compared with “treatment as usual” with regards to hypoglycemic events, with no difference in the rates of level 1 and level 2 hypoglycemia between both the preapp and app-use phases of the study. Hence, “acT1ve” is a safe app that can be used to guide diabetes management during and after exercise. Even though the use of mHealth technologies has become common practice for diabetes self-management [32,33], there are currently no commercially available apps that provide real-time evidence-based advice for managing glucose levels around exercise. The “acT1ve” app has the potential to fill this gap.

The noninferiority and thus safety of the acT1ve app is supported by the finding that hypoglycemia rate and the percentage of time spent both in the target glucose range (3.9 to 10 mmol/L) as well as above the target glucose range (>10 mmol/L) were similar for both the preapp and app-use phases. These are important findings in the context of the noninferiority testing that is required by the Australian regulatory body to make this app reach the market.

Many mobile app–based interventions have been reported to improve glycemic management in diabetes [34]. Here, in contrast, there was no trend for the use of acT1ve to improve blood glucose management before or during exercise. Such a finding is not surprising, particularly in view of the comments made by some of our participants who noted that the acT1ve app was more likely to be beneficial for newly diagnosed individuals or individuals who are new to exercise. This was not the case for any of our participants, as they had been diagnosed with T1D for at least one year and had been exercising regularly at the time they joined the study. Future studies are thus required to examine the benefit of our app in improving glycemic management in people who engage in unpredictable heterogeneous patterns of physical activities or those who experience a sudden transition from an inactive lifestyle to one that is more physically active.

Exposure to the acT1ve app during the App-use phase did not result in any significant changes in exercise frequency, intensity, duration, and workload (Figure 4) compared with the Preapp phase. A plausible explanation for this lack of significant differences between the preapp and app-use phases may be related to the tendency of many individuals to stick to their routine and perform activities that they are more well-versed in or comfortable with. In this respect, it is noteworthy that the average pattern of activity of our participants was similar to that of people without T1D and of equivalent age in Australia [35], with the exception of strength-based activities. Also, our results should be interpreted with caution since the way the exercise data were collected may have masked some small but significant changes between treatment phases. Indeed, data were collected from the combination of available data from the 3 different monitoring platforms (ie, acT1ve, Garmin watch, and paper diary). Also, the calculations to estimate exercise intensity were based on either the use of a MET formula [36] or on predetermined MET values for specific activities outlined16 which are prone to error.

The participant interviews highlighted that participants did not use the app to its full potential. The average duration of diabetes of our cohort was 6.9 years, and most were already very active before starting the study. This suggests that participants were happy with the management plan they already had in place for exercise and used the app only to supplement their current exercise management regime. However, all participants stated that they enjoyed using the app and found the information to be trustworthy and reassuring. All participants felt the app was better suited to individuals who were newly diagnosed or new to exercise. In addition, participants felt the accuracy of the information would encourage those individuals who were reluctant or fearful to engage in exercise, as well as benefit parents and their young children who were growing and navigating diabetes and exercise. In addition, all participants indicated that they would either use the app again or would recommend it to others.

The acT1ve app received good scores for each of the uMARS subscales and its overall quality. The acT1ve app was found to be engaging, usable, informative, and functional with appropriate esthetics. The participants also liked the design of the app. The acT1ve app also compares favorably with the uMARS scores of 89 popular diabetes apps [32]. Indeed, this subset of mobile health apps ranked “acceptable-good” in engagement, functionality, and esthetics, and they ranked “poor-acceptable” in information, app quality score, and app subjective score [32]. Our qualitative analyses also revealed that many participants liked the simple interface of the app, which was easy to navigate, straightforward, and user-friendly. Some of the participants found the information provided by the app to be relevant, appropriate, and clear, with a simple and easy flow of presentation. However, a few found the navigation a little confusing.

Participants’ feedback for future improvements of the app included advice for exercise that lasts longer than one hour, more flexibility in recording the duration of their activity, integration of CGM levels into the app, and facilitation of communication with health care providers. In addition, they suggested the addition of video options for visual learners. Most of these app features desired by the participants were recommended in the initial exercise workshops that we had conducted before developing “acT1ve.” However, these recommendations could not be incorporated into the app design due to the lack of funds.

### Strengths and Limitations

Some of the strengths of the study are both the testing in a free-living setting of an app that is co-designed with young people with T1D and the use of both quantitative and qualitative methodologies to gain an objective and subjective perspective regarding the usability and acceptability of the app. We recognize the various limitations of this study. First, as alluded to earlier, was the lack of a control group, a study design that was adopted to increase the power of our study, but at the expense of its validity. Indeed, the use of a one-arm study design prevents disentangling treatment effects from other effects such as the Hawthorne effect, order effect, regression to the mean, and factors affecting the frequency of physical activity over time. To minimize the impact that these factors might have on our findings, the study was completed during school term to ensure a stable physical activity pattern, and contact with research staff was kept to a minimum. Despite adopting those measures, there were other challenges faced during this study, mainly the COVID restrictions on sports and activities in place in Western Australia, as well as school exams during the intervention phase.

The second limitation was the short duration of the study and small sample size. The study duration of 12 weeks is inadequate to capture seasonal variations in exercise or “novelty waning” effects. Although the sample size and study duration were statistically sufficient for demonstrating noninferiority, a longer trial with a larger cohort could uncover more nuanced effects on glycemic outcomes, exercise habits, and sustained user engagement. To formally test efficacy and benefits of acT1ve and overcome the potential Hawthorne and selection biases of a single-arm study, future randomized controlled trials with a larger sample size and a 6- to 12-month extension will incorporate validated behavior-change measures and comparator arms to assess whether observed improvements reflect true intervention effects or observation biases.

The third limitation was the limited generalizability to less experienced or less active individuals. Most of our participants were physically active and confident in their exercise-related glucose management, and it remains unclear how acT1ve might perform among newly diagnosed individuals. We recognize that adolescents newly diagnosed with T1D or those with sedentary lifestyles may face unique behavior-change challenges (lower baseline self-efficacy and minimal exercise habits). In our future studies, we plan to recruit a more heterogeneous sample across age, activity levels, and years since diagnosis. Since more than half of the cohort did not use the app for their regular exercise, we will include subgroup analysis in future work, stratifying by prior baseline exercise experience and diabetes management proficiency, to determine which subgroups might derive the greatest benefit from the app.

### Conclusions

In summary, despite the limitations inherent to our study design, we conclude that “acT1ve,” a mHealth app which was developed in collaboration with young people with T1D, is safe for diabetes management around exercise. Our findings suggest that our app may play a more important role in helping individuals manage their blood glucose in the face of sudden changes in their pattern of physical activity. Whether this is the case and whether the other possible benefits uncovered by our qualitative data hold true await the performance of a larger-scale randomized control trial to examine the extent to which the use of acT1ve app promotes greater self-efficacy in managing diabetes around exercise.

## Supplementary material

10.2196/68694 Multimedia Appendix 1 Interview questions.

10.2196/68694 Checklist CONSORT checklist.

## Abbreviations

CGM: continuous glucose monitoring
CONSORT: Consolidated Standards of Reporting Trials
CSII: continuous subcutaneous insulin infusion
GI: glycemic index
HbA_1c_: glycated hemoglobin A_1c_
MDI: multiple daily injections
MET: metabolic equivalent of tasks
mHealth: mobile health
REDCap: Research Electronic Data Capture
T1D: type 1 diabetes
uMARS: user Mobile Applications Rating Scale
