# Supplementary material for: A Novel Mobile Health App to Educate and Empower Young Adults With Type 1 Diabetes to Exercise Safely: Prospective Single-Arm Pre-Post Noninferiority Clinical Trial
Source: JMIR Diabetes. 2025 Aug 22;10:e68694. doi: 10.2196/68694 (PMC12373410; doi:10.2196/68694)
Supplement: Multimedia Appendix 1 [file diabetes-v10-e68694-s001.pdf]

Views and experiences of participants trialling T1D Exercise APP

Introducing the Interview

- Now that you have used the Exercise APP we would like to hear about what you **liked and didn't like** about the APP, what you thought of the **features,** and if you think you would **use it**.

- For the purposes of this interview, **exercise** relates to **activity requiring physical effort**, for example, team sports, sport classes, gym sessions, walking to school, mowing the lawn etc.

- There are no right or wrong answers

- The recorded information will not be identifiable, we will not use any names

- This information will be audio recorded but will remain confidential

- Your participation is voluntary so we can stop the interview at any time

- You can choose not to answer any of the questions

- This will take about 20 minutes; you can take a break at any time

- The recorder can be turned off at any time you ask, or parts of the recording can be removed

- Do you have any questions for me?

How often did you exercise during the study period including times you didn't use the APP?

- What types of exercise did you most commonly participate in? (type, duration, intensity)

Was exercise more or less enjoyable using the APP compared to your regular management?

- On a scale of 1 to 5 where 1 = exercise was less enjoyable; 3 = about the same enjoyment; 5 = exercise was more enjoyable
  - So you picked a number (**) – can you tell me why you didn't give it a higher/ lower score?

Did you exercise more or less with the APP compared to your regular management?

- On a scale of 1 to 5 where 1 = exercised less with the APP; 3 = exercised about the same amount; 5 = exercised more with the APP
  - Did you exercise more often, for a longer duration, or both?
  - Did the APP allow more freedom to exercise spontaneously?
  - Did you participate in different types and intensities of sport?

Were you more or less confident to exercise with the APP compared to your regular management?

- On a scale of 1 to 5 where 1 = you were less confident; 3 = about the same level of confidence; 5 = you were more confident
  - So you picked a number (**) – can you tell me why you didn't give it a higher/ lower score?

Did you understand how to use the APP?

- On a scale of 1 to 5 where 1 = I did not understand it....... 5 = I did understand
    - So you picked a number (**) – can you tell me why you didn't give it a higher/ lower score?
    - Did you feel confident using the APP?
    - How long did it take you to feel confident using the app?
    - Were there enough prompts or instructions?
    - Looking back what would you have liked to have known from the start?

How much did you trust the APP?

- On a scale of 1 to 5 where 1 = did not trust the APP at all; 5 = completely trusted the APP
    - How often did you exercise with the APP?
    - When you did not use it, why didn't you use it?
        - Prompts: was it due to not being suitable for the sport or because you did not trust it?

How often did you follow the advice the APP gave?

- On a scale of 1 to 5 where 1 = did not follow the advice at all; 5 = always followed the advice
    - On the times you chose to do something else, why was this?

Was the information you entered into the APP accurate?

- On a scale of 1 to 5 where 1 = never accurate at all; 5 = always accurate
    - So you picked a number (**) – can you tell me why you chose this score?

Views and experiences of participants trialling T1D Exercise APP

Did you stop using the APP before the end of the 4 weeks?

- If YES: What was the reason you stopped using it?

Overall what did you think of the APP?

- On a scale of 1 to 5 where 1 = it's awful…………… 5 = it's great
  - So you picked a number (**) – can you tell me why you didn't give it a higher/ lower score?

How likely are you to recommend this APP to others with type 1 diabetes?

- On a scale of 1 to 5 where 1= not likely …… 5 = very likely
  - Explore score

If you had the opportunity to use the APP again,

- Would you use it?
- How long do you think you would use it for?
- Would you use it all the time or just during certain times/situations?
- If during certain times/situations, what are these?

Ending the interview

These were all the questions I had for you. Do you have any question for me or anything you would like to add to this interview?

Thank you for taking part.
